# Liquid Pressure Sensor Based on Fiber Bragg Grating with an Adjustable Structure

**DOI:** 10.3390/s22239188

**Published:** 2022-11-26

**Authors:** Junda Lao, Chao Wang, Yaqi Tang, Pengfei Zheng, Liuwei Wan, Chi Chiu Chan, Shuangchen Ruan

**Affiliations:** Center for Smart Sensing System, Julong College, Shenzhen Technology University, Shenzhen 518118, China

**Keywords:** adjustable, FBG, liquid pressure, PDMS

## Abstract

In this paper, a fiber-optic liquid pressure sensor is designed and developed by encapsulating the fiber Bragg grating (FBG) inside the adjustable double-flange cylinder rigid structure with flexible polymer polydimethylsiloxane (PDMS). Within the elastic deformation range of the PDMS, the proposed adjustable FBG-based liquid pressure sensor is proven to change its measuring range while maintaining high measurement sensitivity by simply adjusting the structure, that is, the sensor can achieve high measurement sensitivity in various liquid levels. In addition, the simulation and experimental results show that the sensor sensitivity can be enhanced by the proper changes of the structural parameters, such as the inner diameter, etc. The proposed sensor has shown that it has good linearity and stability, which provides a new opportunity for the monitoring of liquid pressure in oceans, dams and other environments.

## 1. Introduction

The acquisition of hydraulic values is of great significance for exploring liquid environments such as oceans, dams and oil wells, etc. At present, the mainstream approach to measuring liquid pressures is through electrical sensors [1,2,3]. However, electrical sensors have limited applications in some liquid environments, because of the effect of electromagnetic interference, difficult signal transmission and safety hazards [4,5]. The fiber-optic sensor is an optical passive element, which has incomparable advantages over electrical counterparts in complicated liquid environment [6,7]. Firstly, fiber-optic probes are flexible, small in diameter and resistant to water and corrosion, which can greatly enhance their lifetime and lessens ecological damage. Secondly, light is used as the signal element inside the fiber (e.g., silica) which is immune to electromagnetic interference, so this is suitable for operation in harsh conditions. Such merits enable fiber-optic liquid pressure sensors to replace electrical sensor in certain situations.

In recent years, the reports on the fiber-optic hydraulic sensing technology mainly included the Michelson interferometer (MI) [8], Fabry–Perot interferometer (FPI) [9] and fiber Bragg grating (FBG), etc. Compared with other structures, the MI-based sensors and the FPI-based sensors are more measurement-sensitive to liquid pressure, but they have some drawbacks, such as narrow measurement range and high requirements for the intensity and stability of the light source and their fragile structures. These make them mainly applicable to micro pressure measurement [10]. Literature on FBG hydraulic-sensing technology has been reported as early as 1993 [11]. Although the measurement sensitivity of the FBG is lower than the MI or FPI, it possesses stable performance, low requirements for light source specifications, simple signal demodulation, low cost, quasi distributed measurement, better resolution and a wide measuring range [12,13]. These make the FBG-based sensors more applicable in practical usages. Most researchers have begun to integrate the FBG with mechanical structures or chemical materials, hoping to fabricate a practical FBG-based liquid pressure sensor with higher measurement sensitivity and ultra-durable structure [14,15].

In 2020, Mingyao Liu et al. [16] reported an immersion liquid pressure sensor, which was mainly made by encapsulating the FBG with the epoxy resin in a specific metal shell; the measurement sensitivity was about 0.0513 nm/MPa within the liquid pressure range of 0–15.5 MPa. That structure offered a wide measurement range but was less sensitive, which greatly increased the cost of signal demodulation. In 2021, Schenato, Luca et al. [17] fabricated a new FBG-based liquid pressure sensor by encapsulating the FBG inside an aluminum alloy waterproof sealing shell with the mechanical structure of a hexagonal pantograph. The measurement sensitivity was up to 240 nm/MPa in the liquid pressure range of 0–0.01 MPa. Although this sensor had a high sensitivity, its measuring range was narrow, and it needed to be sealed in a waterproof structure. These reasons mean it cannot be applied in some practical situations such as oceans or oil wells. Although there have been fruitful results achieved in FBG-based liquid pressure sensors up to now, the co-existence of high sensitivity and wide measuring range has not been solved. Therefore, a new designed liquid pressure sensor is proposed, by encapsulating the FBG inside the adjustable double-flange cylinder mechanical structure where the flexible material PDMS is filled. To a certain extent, the sensor can achieve high measurement sensitivity in various liquid levels by simply adjusting the structure. The static stress analysis and optimization design of the sensor structure are performed by using the COMSOL Multiphysics software. The sensors have been fabricated based on the simulation results and the performance have been verified by experiments. The rest of this paper is structured as follows: the sensor details, such as sensing principle, static stress analysis and packaging process are described in Section 2, and the experimental details, results and discussion are given in Section 3. Finally, conclusion is given in Section 4.

## 2. Details of the Sensor

### 2.1. Sensing Principle

**Figure 1:**
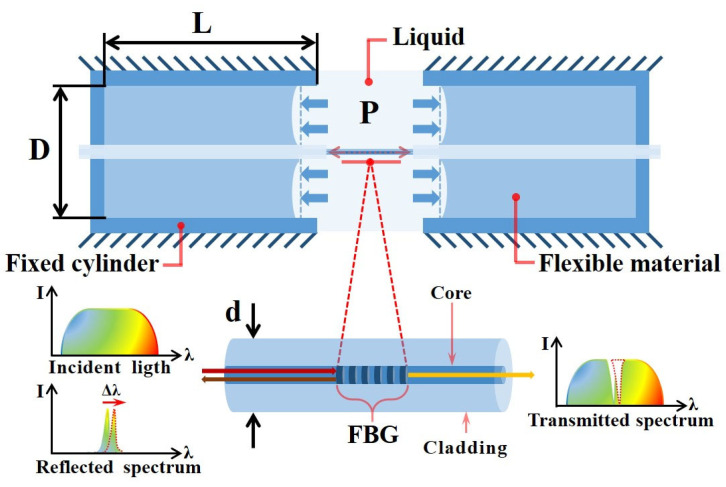
Model of the FBG-based liquid pressure sensor.

Figure 1 shows the model of the proposed FBG-based liquid pressure sensor. Both ends of the FBG are tightly connected with the flexible material which is sealed in the inner part of the fixed cylinders. From Figure 1, it is known that the flexible material is in contact with liquid and acts as a pressure transducer, which can convert an external liquid pressure on its surface into a longitudinal strain on the FBG through the elastic deformation of the material. Thus, the flexible material should have the characteristics of non-toxic, high elastic deformation, no chemical reaction with the measured liquid environment and high adhesion with the optical fiber surface, etc. When the FBG is sensitive to the longitudinal strain, the corresponding Bragg wavelength will be changed accordingly with the change of the strain effect on the FBG. Finally, the hydraulic pressure can be determined by simply measuring the Bragg wavelength variation accordingly.

As shown in Figure 1, supposing that the flexible material is elastically deformed in its longitudinal direction and the frictional coefficient between flexible material and the inner surface of fixed cylinder is neglected, the structure can be understood as a simplified piston-air cylinder model. Since the both ends of the FBG are tightly immersed with the filled flexible material inside the fixed cylinders, the longitudinal strain on the FBG Δε is formed by the elastic deformation of flexible material expressed as:

(1) $$\Delta \varepsilon =\frac{D^{2}}{Ed^{2}}\Delta p$$

where D is the inner diameter of the sensor, d is the diameter of the fiber cladding, E is the Young’s modulus of optical fiber and Δp is the variation of liquid pressure.

It is well known that when the ambient temperature of the FBG is constant, the relationship between the initial Bragg wavelength of the FBG $\lambda_{B}$ and the Δε is expressed as [18]:

(2) $$\frac{\Delta \lambda_{B}}{\lambda_{B}}=\left(1-p_{e}\right)\Delta \varepsilon$$

where $\Delta \lambda_{B}$ is the Bragg wavelength variation caused by the strain, $p_{e}$ is the photo-elastic coefficient of the fiber core, about 0.22 [11]. The sensor sensitivity is the ratio of the $\Delta \lambda_{B}$ to the Δp, and the following is true based on Equations (1) and (2).

(3) $$\frac{\Delta \lambda_{B}}{\Delta p}=\frac{\left(1-p_{e}\right)D^{2}}{Ed^{2}}\lambda_{B}$$

Since the values of d, E, $\lambda_{B}$ and $p_{e}$ are constants, the sensor sensitivity $\frac{\Delta \lambda_{B}}{\Delta p}$ is directly proportional to the internal cross-sectional area of the sensor.

### 2.2. Analysis on the Structure Parameters

In this work, the PDMS (SYLGARD 184, Dow Coming Corporation, Midland, MI, USA) is the best flexible material we have found for the sensor. As a high molecular silicone compound, the PDMS possesses a low elastic modulus, low moisture absorption, low toxicity and strong adhesion to the surface of optical fiber after curing, which is made by mixing the base and the curing agent according to a certain mass ratio. When the mass ratio is 10:1 (base: curing agent), the elastic modulus of the PDMS is 1.628 MPa [19]. The cylinders of the sensor were made of stainless steel with high stiffness, of which deformation was hardly affected by external hydraulic pressure. The length of the FBG was 6 mm in this simulation. According to Equation (3), the inner diameter of the sensor (D) was the key parameter affecting the sensor sensitivity. Therefore, with the other parameters fixed, such as the temperature and ratio of inner diameter to length of cylinder (10:23), the inner diameter of the sensor in various sizes were subjected to simulation by using COMSOL Multiphysics to investigate the effects of the D on the measurement sensitivity. Furthermore, in order to ensure that the simulation model was valid, it was necessary to consider the adhesion, the slippage and the friction coefficient between various materials as much as possible.

**Figure 2:**
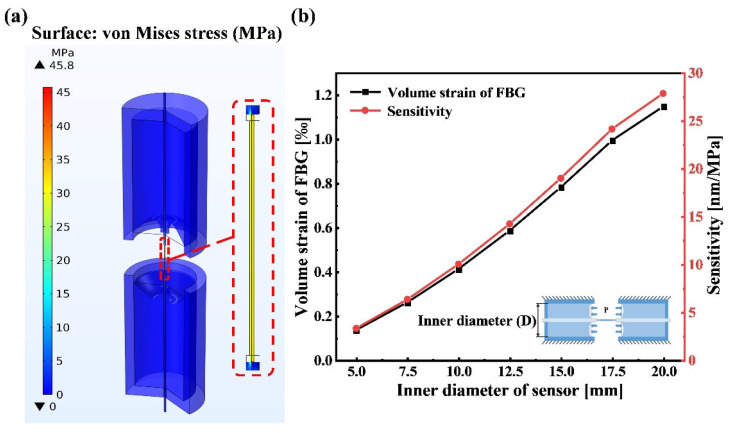
Modal analysis: (**a**) static stress analysis of sensor with 10 mm inner diameter; (**b**) the measurement sensitivities of sensor with various inner diameter sizes in simulation.

A fixed constraint was applied to the outside surface of the sensor with a 10 mm inner diameter, while 0.05 MPa external pressure was applied to the end surfaces of the PDMS (10:1) to obtain the equivalent stress cloud of the sensor model as shown in Figure 2a. From Figure 2a, we can see the maximum deformation from the PDMS in the sensor, and that the volume strain on the FBG was caused by the stretching of PDMS on both sides. The strain effects on the FBG in various D and the corresponding sensor sensitivities are shown in Figure 2b. From Figure 2b, it indicates that the sensor sensitivity increases as the D increases.

### 2.3. Fabrication

**Figure 3:**
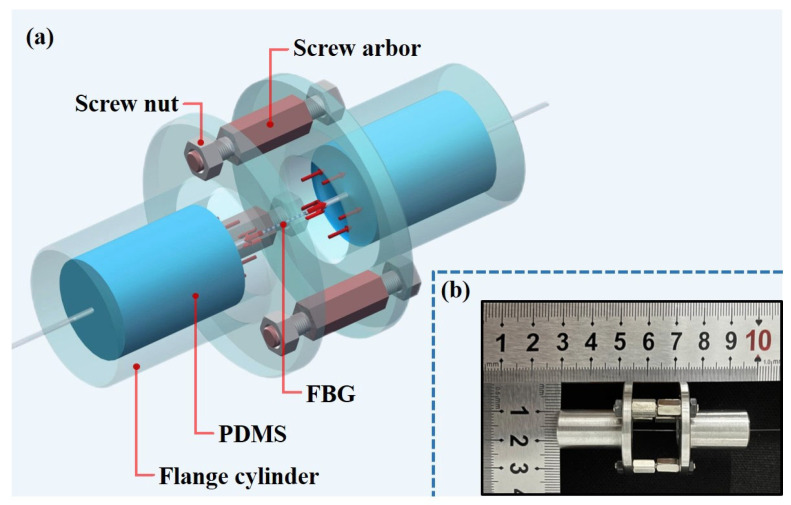
Sensor structure: (**a**) 3D schematic diagram of the sensor; (**b**) sensor prototype.

In order to fabricate a practical FBG-based liquid pressure sensor, the fixed cylinders in Figure 1 are replaced by two general flange cylinder parts. Figure 3a shows the 3D schematic diagram of the sensor. As shown in the sensor diagram, the two-flange cylinder parts are assembled and fixed by the screw arbors and nuts, the PDMS are filled into the two flange cylinders and both ends of the FBG are closely immersed with the PDMS. Figure 3b shows the sensor prototype with 10 mm inner diameter. From Figure 3, it is known that the size of components and parts such as the flange cylinders, the screw arbors and nuts affect the overall dimensions of the sensor. Therefore, the overall dimensions of the sensor can be controlled by changing the size of components and parts.

**Figure 4:**
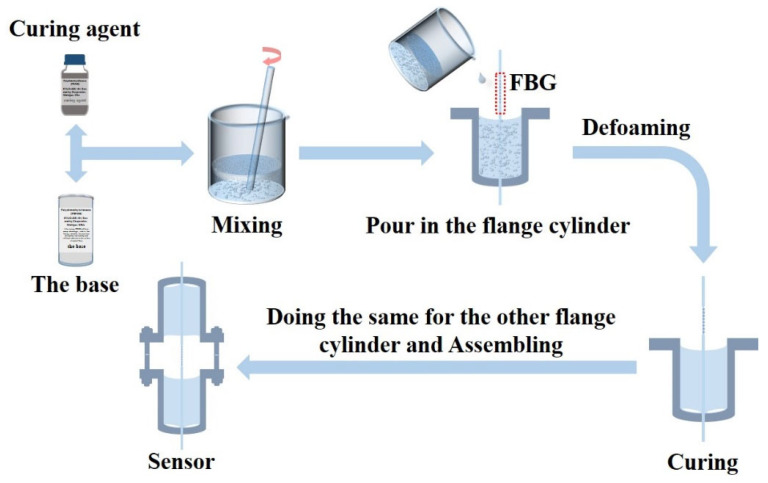
Fabrication process of the proposed sensor.

The fabrication steps of the proposed sensor are described as follows and shown in Figure 4. (1) The PDMS colloid can be obtained by mixing the base with a curing agent in a mass ratio of 10:1 and fully stirring with a glass rod for 15 min. (2) The FBG with a fiber pigtail is passed through the central hole at the bottom of one flange cylinder which is fixed on the base of the fixture, and the FBG is located above the flange cylinder. (3) The PDMS colloid is poured in the inner part of flange cylinder to cover the fiber but the FBG is exposed to air. (4) The flange cylinder with the PDMS colloid FBG is placed in the cold storage room at −19 °C for 9 h or more, so that the bubbles in the PDMS colloid can be removed naturally. (5) The semi-finished sample is taken out from the cold storage room and placed in a room temperature environment for 48 h or more until the PDMS in the flange cylinder is fully cured and closely connected with the fiber surface. (6) The screw arbors and the nuts are assembled to the flange cylinder assembly hole, respectively, and the packaging process is repeated for another flange cylinder to the other end of FBG’s pigtail fiber. To summarize, the time for the fabrication of the sensor is about 5 days.

## 3. Results and Discussion

**Figure 5:**
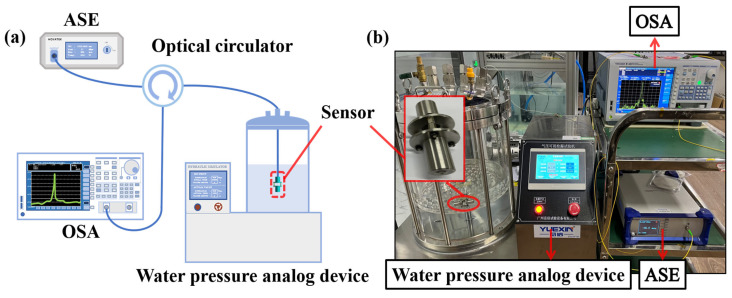
Experiment: (**a**) schematic diagram of the sensor testing system; (**b**) experimental setup.

As shown in Figure 5a, the fabricated sensor (Figure 4) was connected to an amplified spontaneous emission source (ASE) and an optical spectrum analyzer (OSA) through an optical fiber circulator. Afterwards, the sensor was placed in the water pressure analog device which can simulate the water level between 0 m and 50 m by applying pressure (from 0 MPa to 0.5 MPa). The characteristics of the sensor can be examined, such as sensitivity, linearity and stability to water pressure. In this investigation, due to the sensor being sensitive to the liquid temperature and the pressure simultaneously, the water temperature was kept at 26 °C and monitored by a thermocouple device in order to eliminate the interference of temperature during the experiments. Figure 5b shows the experimental setup.

**Figure 6:**
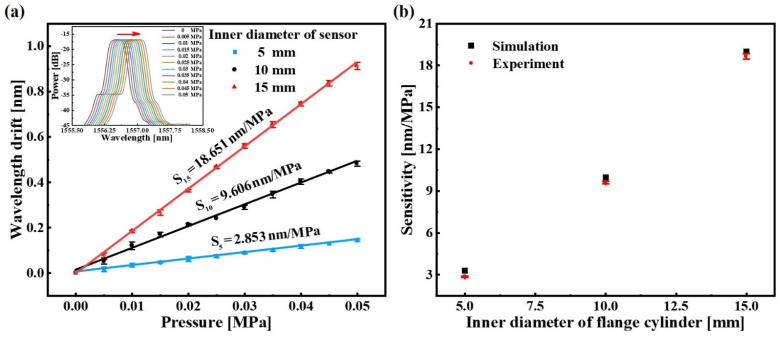
The liquid pressure testing results of the sensors: (**a**) linear fitting results of the sensors with inner diameters of 5 mm, 10 mm and 15 mm; (**b**) comparison of experimental results and simulation results of the sensors. The error bar is obtained by critically repeating the experiment of liquid pressure measurement three times.

The performances of the sensors with 5 mm, 10 mm and 15 mm inner diameter in the measurement range of 0 m to 5 m water level were tested, respectively. At a constant water temperature of 26 °C, the water level was increased from 0 m to 5 m at steps of 0.5 m. For investigating repeatability, the sensor was subjected to three sets of pressure increment experiments. The liquid pressure testing results of the sensors are shown in Figure 6. From the inset diagram of Figure 6a, as the liquid pressure around the sensor increases, the reflection spectrum of the sensor output shows a red shift. Moreover, the higher the liquid pressure, the greater variation of the Bragg wavelength. It proves that the sensor can convert the external liquid pressure into the Bragg wavelength variation due to the elastic compression deformation of the PDMS inside the cylinders. The wavelengths of these offset peaks are extracted and subjected to a linear fitting, and the relationships between the wavelength drifts of the sensors and the liquid pressures are shown in Figure 6a. From the error bar and the linear fitting outcomes, it can be observed that all these sensors have good linearity and the measurement sensitivity increases as the inner diameter of the sensor increases. The sensor with 15 mm inner diameter has the highest measurement sensitivity, about 18.651 nm/MPa, and the coefficient of fitting determination is R^2^ = 0.9994. Figure 6b shows the simulation results and experimental results of the sensor, and the sensors’ sensitivities in experiments are slightly smaller than in the simulations. The discrepancy is that the simulation results are obtained under the condition that various parameters tend to be idealized, but in the experiments, the results are often affected by the errors of the sensor parameters and the errors of the instruments. Furthermore, the measurement accuracy of the sensor is mainly evaluated by the instrument’s accuracy, such as the OSA and the water pressure analog device.

**Figure 7:**
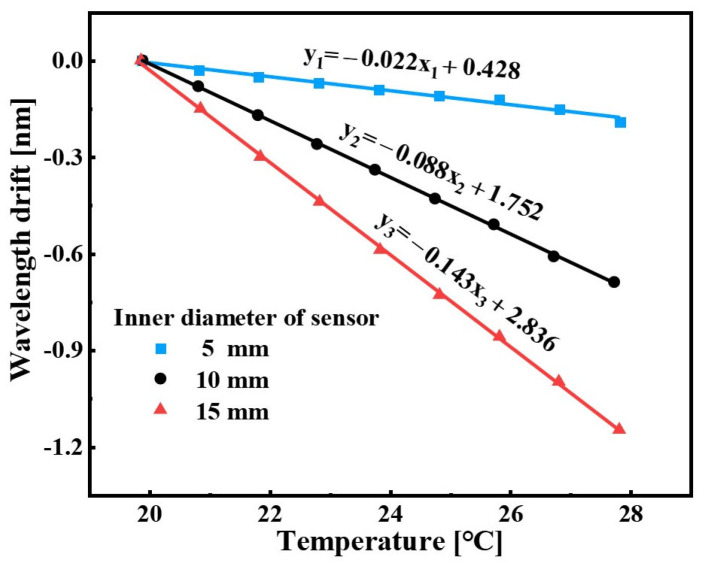
Temperature effect on the sensor.

It is well known that the FBG is sensitive to both the temperature and strain. In order to guarantee the stability of the sensors in engineering applications, the temperature characteristic of the sensors was calibrated by the water-bath heating device and the thermocouple. The water temperature in the water-bath heating device was increased from 20 °C to 28 °C, continuously maintained for 10 min in each step, and monitored by the thermocouple. The linear fitting outcomes between the peak wavelength shift of the sensors and the water temperature are shown in Figure 7. From the results, the temperature sensitivities of the sensors with 5 mm, 10 mm and 15 mm are −0.022 nm/°C, −0.088 nm/°C and −0.143 nm/°C, respectively, and the corresponding hydraulic measurement errors caused by the liquid temperature are 0.008 MPa/°C, 0.009 MPa/°C and 0.008 MPa/°C, respectively. The results after comprehensive analysis indicated that the response of the sensor to temperature was due to the thermal expansion and contraction effect of the PDMS in the flange cylinders. Although the sensors are less affected by the ambient temperature, in order to further eliminate the temperature interference, the FBG-based sensing configuration can be used to measure temperature and liquid pressure simultaneously. For example, the other FBG sensor is encapsulated inside the PDMS to form an independent temperature-sensing unit.

According to Equation (3), it is found that the theoretical sensitivities of the sensors with 5 mm, 10 mm and 15 mm inner diameters are 26.565 nm/MPa, 106.259 nm/MPa and 239.083 nm/MPa, respectively, which are each about 10 times the sensitivities of the experiments. The discrepancy is mainly due to that the PDMS cannot completely convert the liquid pressure into the in-line strain on the FBG. Firstly, the PDMS will store kinetic energy like a spring model when it is compressed and relaxed. Secondly, the PDMS colloid adheres to the inner wall of flange cylinder and has a lot of friction with the inner wall after curing, so that the longitudinal elastic deformation of the PDMS is reduced. Thirdly, there is slight slippage between the PDMS and the fiber surface because of the small contact area and the limited adhesion. Further, the limited longitudinal elastic deformation of the PDMS and the limited adhesion between the PDMS and the optical fiber surface are also the reasons for the limitation of the sensing depth.

There are several methods that can effectively improve the measurement sensitivity by modifying the structure properties, as follows. Firstly, the flexible material with stronger adhesion to the fiber surface can be used to replace the PDMS, or a waterproof curing agent with higher viscosity can be applied between the PDMS and fiber surface. Secondly, the adhesion and the friction between the polymer and inner wall of the flange cylinders need to be reduced. Thirdly, the longitudinal elastic deformation of the PDMS is increased by increasing the length of flange cylinders (L), decreasing the elastic modulus of the PDMS (E) or forming air cavities between the PDMS and the bottom of flange cylinder through a syringe.

**Figure 8:**
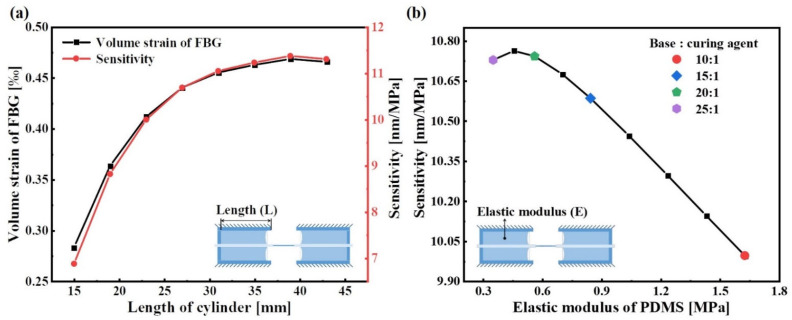
Parameter analysis of the sensor with 10 mm inner diameter: (**a**) the relationship between the measurement sensitivity and the length of the cylinder; (**b**) the relationship between the measurement sensitivity and the elastic modulus of the PDMS.

The simulations for investigating the effects of the L and the E on the sensor measurement sensitivity were undertaken. Figure 8 shows the simulation results of the sensor with a 10 mm inner diameter in the case of the various L and E, respectively. From Figure 8a, with the other parameters fixed, it can be observed that the measurement sensitivity increases as the L increases, but the improvement is limited. When L is 39 mm, the measurement sensitivity of the sensor with 10 mm inner diameter is the highest, about 11.375 nm/MPa. From Figure 8b, with the other parameters fixed, the measurement sensitivity is negatively correlated with the E. According to the experimental results of Justin D. Glover, it is known that the elastic modulus of the PDMS decreases as the mass ratio of the base and the curing agent increases [19]. That is, the PDMS with elastic modulus of 1.628 MPa, 0.845 MPa, 0.561 MPa and 0.351 MPa were made by the mass ratio of 10:1, 15:1, 20:1 and 25:1 (base: curing agent), respectively. Therefore, the measurement sensitivity can be improved by increasing the mass ratio of the base and the curing agent. From Figure 8b, when the mass ratio of the base and the curing agent in the PDMS is 20:1, the sensor with 10 mm inner diameter has the highest measurement sensitivity, about 10.742 nm/MPa. In addition, it was found from the simulation results that the measurement sensitivity of the sensor with the air cavity between the PDMS and the bottom of cylinder would be greatly improved. Therefore, the measurement sensitivity can also be improved by the injection of more air cavities between the PDMS and the bottom of the flange cylinder by the syringe.

**Figure 9:**
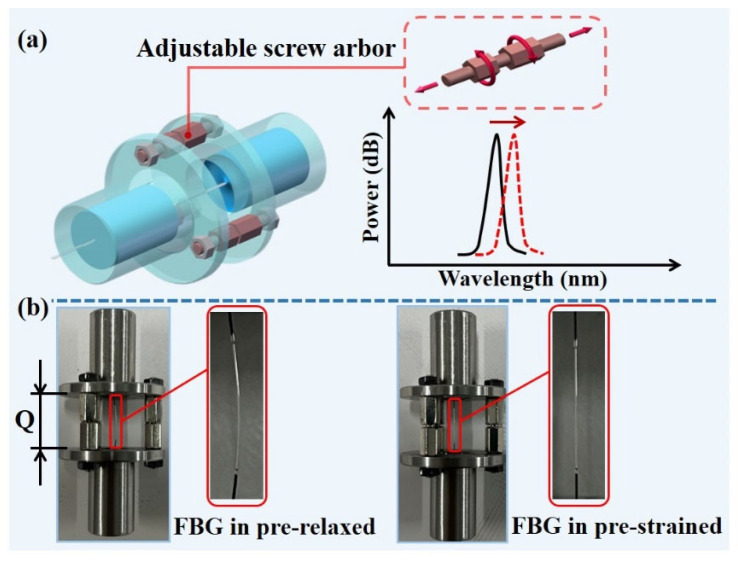
The sensor with adjustable structure: (**a**) 3D schematic diagram of the sensor; (**b**) different FBG states after adjusting the screw arbors.

For extending the measurement range with high measurement sensitivity, the structurally adjustable FBG-based liquid pressure sensor is proposed and shown in Figure 9. From Figure 9a, the initial Bragg wavelength of the FBG in the sensor can be changed by simply adjusting the screw arbors. Figure 9b shows the pre-relaxed and pre-strained state of the FBG after adjusting the screw arbor, respectively. Therefore, by adjusting the sensor structure to change the state of the FBG, the sensor can be kept at a high measurement sensitivity in various water levels. For example, when the sensor is used to measure the liquid pressure in shallow coastal water, pre-strained FBG is suitable. On the other hand, when the sensor measures the liquid pressure in deeper water levels, pre-relaxed FBG is needed.

**Figure 10:**
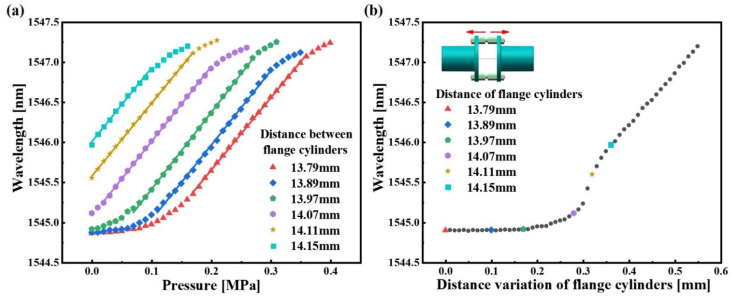
Experimental results: (**a**) the measuring range of sensor corresponding to different distance between flange cylinders; (**b**) the relationship between the distance variation of flange cylinders and the initial Bragg wavelength of the sensor.

The sensor with 10 mm inner diameter is here used as an example. Figure 10a shows the measurement range of the sensor under different $\lambda_{B}$. From Figure 10a, it can be observed that the measurement range of the sensor varies with the distance between the flange cylinders (Q). When Q is 13.79 mm, 13.89 mm, 13.97 mm or 14.07 mm, the FBG in the sensor is in pre-relaxed state, and the corresponding sensor measurement sensitivities are 9.035 nm/MPa, 9.318 nm/MPa, 9.595 nm/MPa and 9.342 nm/MPa in the measurement range of 0.17 MPa to 0.36 MPa, 0.11 MPa to 0.3 MPa, 0.07 MPa to 0.27 MPa and 0.02 MPa to 0.2 MPa liquid pressure, respectively. On the other hand, when Q is 14.11 mm and 14.15 mm, the FBG in the sensor is in a pre-strained state, the corresponding measurement sensitivities are 9.105 nm/MPa and 9.206 nm/MPa in the measurement range of 0 MPa to 0.17 MPa and 0 MPa to 0.1 MPa liquid pressure, respectively. From the linear fitting outcomes, it can be shown that the sensor has high measurement sensitivity and good linearity in various water levels.

In order to improve the feasibility of the scheme, the relationship between the distance variation of flange cylinders (ΔQ) and the $\lambda_{B}$ should be studied and determined. In this work, the tensile testing machine and the OSA were used to determine the relationship between ΔQ and the $\lambda_{B}$. The two sides of the sensor were fixed on the fixed clamp and the movable clamp of the tensile machine, respectively. The stretching range of the tensile machine was set to 0–0.55 mm at a step size of 0.01 mm. Then, the relationship between ΔQ and the $\lambda_{B}$ of the sensor is shown in Figure 10b. From the result, when ΔQ varies from 0 to 0.19 mm, the $\lambda_{B}$ of the sensor is almost unchanged. When ΔQ varies from 0.19 to 0.55 mm, the $\lambda_{B}$ of the sensor increases as ΔQ increases. Thus, the proposed sensor can maintain high sensitivity in various water levels by adjusting the structure and the quasi-distributed liquid pressure sensing system with high sensitivity and large measurement range can be established by fully combining the advantages of the adjustable structure of the sensor and the quasi-distributed property of the FBG.

## 4. Conclusions

In this study, the adjustable structure FBG-based liquid pressure sensor is proposed and its feasibility and performance are verified by simulation analysis and experiments. The following two conclusions are obtained from the findings. Firstly, the measurement sensitivity is positively correlated with the inner diameter of the sensor. The measurement sensitivity of the sensor with a 15 mm inner diameter was 18.651 nm/MPa. According to the simulation analysis, the sensors for higher sensitivities can be designed by properly changing its structural parameters, such as the cylinder length and the PDMS elastic modulus, etc. Secondly, we propose the adjustable structure sensing model which is capable of the co-existence of high measurement sensitivity and the wider measurement range, to a certain extent. On the whole, the sensor is simple to fabricate, stable in performance and has excellent sensitivity and linearity, which provides a new way for monitoring liquid pressure or level in oceans, oil wells, etc.

## Data Availability

Not applicable.
